# Burden of Human Papillomavirus among Haitian Immigrants in Miami, Florida: Community-Based Participatory Research in Action

**DOI:** 10.1155/2012/728397

**Published:** 2012-03-15

**Authors:** Erin Kobetz, Jonathan K. Kish, Nicole G. Campos, Tulay Koru-Sengul, Ian Bishop, Hannah Lipshultz, Betsy Barton, Lindley Barbee

**Affiliations:** ^1^Department of Epidemiology and Public Health, University of Miami Leonard Miller School of Medicine, Clinical Research Building, Suite 1033 1120 NW 14th Street, Miami, FL 33136, USA; ^2^Division of Cancer Prevention and Control, University of Miami Sylvester Comprehensive Cancer Center, Miami, FL 33136, USA; ^3^Jay Weiss Center for Social Medicine and Health Equity, University of Miami Miller School of Medicine, Miami, FL 33136, USA; ^4^Center of Excellence for Health Disparities Research: El Centro, School of Nursing and Health Studies, University of Miami, Miami, FL 33146, USA; ^5^Department of Medicine, University of Miami Miller School of Medicine, Miami, FL 33136, USA; ^6^Department of Medicine, University of Washington School of Medicine, Seattle, WA 98195, USA

## Abstract

*Background*. Haitian immigrant women residing in Little Haiti, a large ethnic enclave in Miami-Dade County, experience the highest cervical cancer incidence rates in South Florida. While this disparity primarily reflects lack of access to screening with cervical cytology, the burden of human papillomavirus (HPV) which causes virtually all cases of cervical cancer worldwide, varies by population and may contribute to excess rate of disease. Our study examined the prevalence of oncogenic and nononcogenic HPV types and risk factors for HPV infection in Little Haiti. *Methods*. As part of an ongoing community-based participatory research initiative, community health workers recruited study participants between 2007 and 2008, instructed women on self-collecting cervicovaginal specimens, and collected sociodemographic and healthcare access data. *Results*. Of the 242 women who contributed adequate specimens, the overall prevalence of HPV was 20.7%, with oncogenic HPV infections (13.2% of women) outnumbering nononcogenic infections (7.4%). Age-specific prevalence of oncogenic HPV was highest in women 18–30 years (38.9%) although the prevalence of oncogenic HPV does not appear to be elevated relative to the general U.S. population. The high prevalence of oncogenic types in women over 60 years may indicate a substantial number of persistent infections at high risk of progression to precancer.

## 1. Introduction

Haitian immigrant women residing in Little Haiti, a large ethnic enclave in Miami-Dade County, experience the highest cervical cancer incidence rates in South Florida. Between 2007–2009, disease incidence in Little Haiti (34 per 100,000 women) was nearly four times higher than that reported for the Miami metropolitan area overall (9 per 100,000 women) [1]. This disparity reflects lack of access to the formal healthcare system and screening with cervical cytology (Papanicolaou test) [2, 3]. While cytology-based screening programs have effectively reduced cervical cancer incidence and mortality in the United States [4, 5], Haitian women in Little Haiti encounter multiple barriers to routine Pap testing, and often cannot comply with screening recommendations or necessary follow up for detected abnormalities [6, 7].

Underutilization of screening may not solely account for the excess burden of cervical cancer observed among Haitian women. Epidemiologic and virologic studies have shown that persistent cervical infection with oncogenic “high risk” human papillomavirus (HPV) types cause virtually all cases of cervical cancer worldwide [8], but the prevalence of HPV varies by population. Knowledge of the burden of HPV by type and age will be necessary to establish the utility of vaccines against HPV infection and for the effective design of screening protocols with HPV DNA testing, which may improve uptake of screening in populations with access barriers if self-sampling of HPV DNA specimens proves acceptable and feasible. Using a community-based participatory research (CBPR) approach, our primary objective was to document the prevalence and type distribution of HPV in Little Haiti, which to date has not been described. We also examined risk factors associated with oncogenic HPV infection in our sample.

## 2. Methods

### 2.1. Overview of *Patnè en Aksyon*

The current study was conducted as part of an ongoing CBPR initiative in Little Haiti, which has been described in previous publications [2, 3, 9]. Briefly, CBPR is a research methodology, increasingly popular in the field of public health, which invites community participation throughout the research process, from study conceptualization to dissemination of findings [10–12]. This approach helps dissuade community suspicion about the intent of inquiry, which is prevalent in Little Haiti and other underserved communities that are largely disenfranchised from the formal healthcare system.

In Little Haiti, CBPR efforts are governed by a campus-community partnership known as *Patnè en Aksyon *(Partners in Action). This partnership, which involves active participation of community leaders from Little Haiti and an interdisciplinary team of investigators from a large university in the Miami metropolitan area, strives to reduce the excess burden of cervical cancer experienced by Haitian women and to improve the health status of women in Little Haiti. To this end, community health workers (CHWs), who are formally employed by a large community-based organization whose leadership is active in Patnè en Aksyon, play a central role.

### 2.2. Participant Recruitment and Data Collection

In the present study, female CHWs of Haitian descent (fluent in English and Haitian Kreyol) were trained to recruit study participants and collect data using a standardized manual created by one of the academic partners. As part of the training, each CHW also completed an online certification program for conducting human subjects research (CITI), as mandated by the University of Miami's Institutional Review Board (IRB). The university IRB approved the study. 

Between September 2007 and March 2008, CHWs recruited participants primarily through the extensive network of the community-based organization (CBO) where they were formally employed, and by canvassing community venues including flea markets, health clinics, and laundromats across Little Haiti to identify women meeting study eligibility criteria (i.e., were 18 years of age and older, had no prior history of cervical cancer or surgical hysterectomy, and reported having no Pap smear within the past year). The CHWs approached all women in such venues who appeared to be of Haitian descent and at least 18 years of age (*n* = 362) and told them about the study. For women who were interested and eligible (*n* = 290), the CHWs scheduled an interview to have women self-sample for HPV and respond to a short survey.

**Figure 1:**
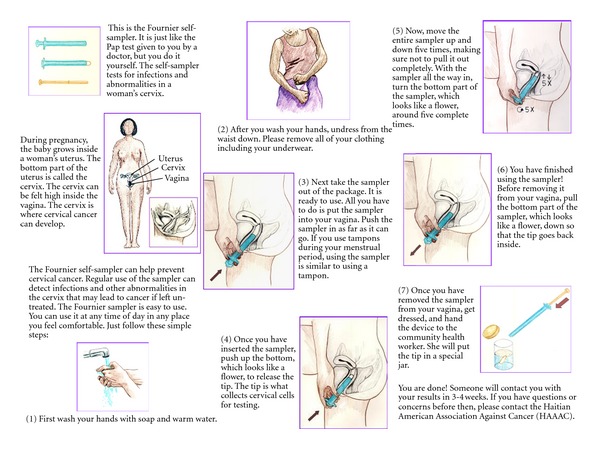
Self-sampling device brochure.

Interviews and self-sampling took place wherever the participant felt most comfortable, usually at her home or the home of a close friend, and were conducted in English or Haitian Kreyol according to the participant's preference. The CHWs were instructed to (1) obtain informed consent, (2) teach women how to appropriately self-sample using visual aids, and (3) interview participants about their experience with self-sampling. To monitor adherence to this research protocol, CHWs were required to log the time that they completed each step on data collection forms. Given widespread skepticism about research in Little Haiti, the CHWs spent one hour (on average) explaining to participants the benefits of participation, explaining the purpose of collecting specimens and genotyping those positive for HPV, and assuring women that results would be kept confidential. Following informed consent, CHWs instructed women on how to appropriately use the device using a pictorial brochure that visually demonstrated each step in the process (Figure 1). The participants then collected their sample in private and gave it to the CHW. Immediately afterwards, the CHW administered a brief questionnaire, which assessed participant's impressions about self-sampling for HPV sociodemographic background, Pap smear screening history, and risk factors for HPV. Questionnaire items were adapted from previously validated women's health behavior surveys and were translated and back-translated from English to Haitian Kreyol for monolingual Kreyol participants.

The CHWs notified all participants of their results, and assisted those with cytological abnormalities and/or infections (e.g., candida, gardnerella, trichomoniasis) in obtaining timely and appropriate followup care. Nearly all women with abnormalities obtained follow-up at a free gynecologic oncology clinic in Little Haiti with the assistance of CHWs.

### 2.3. Specimen Collection

Women self-collected cervicovaginal specimens with the Fournier device, which has demonstrated high concordance with physician-collected cervical specimens and can provide specimens for cytology assays [13, 14]. This device is currently only approved for research purposes, but is under review by the FDA for clinical application. Mechanically similar to a tampon, the device includes an outer sheath to prevent cross-collection of unwanted vaginal cells that may compromise specimen quality and reliability. Women were instructed to insert the device into their vagina, eject the Dacron tip to obtain a sample of cervical cells for cytology, and then retract the tip to avoid cross-collection of vaginal cells during removal (Figure 1). Specimens were shipped to Select Diagnostics (Greensboro, North Carolina) for HPV testing, genotyping, and cytological evaluation. Cytology was performed using standard Thin Prep technology [15].

### 2.4. Human Papillomavirus Detection and Typing

Cervicovaginal specimens were genotyped using a polymerase chain reaction restriction fragment length polymorphism (PCR-RFLP) assay (*Access Genetics*, Eden Prairie, MN), which amplifies the highly conserved L1 region of the HPV genome to detect approximately 80 HPV types. Genomic DNA was extracted from ecto- or endocervical epithelial cells obtained via the Fournier device and fixed in alcohol-based liquid ThinPrep™ (Cytyc Inc, Boxborough, MA) solution using standard techniques. In brief, cellular material was concentrated and the alcohol preservatives removed by distilled water dilution. Each sample was evaluated for cellularity to determine the dilution volume that equalizes the concentrations across all samples followed by an enzymatic incubation to achieve increased cell membrane permeability. An aliquot of each cellular sample was then combined with Cellerate *Access Genetics*, Eden Prairie, MN. This mixture was subjected to thermal cycling incubation resulting in protein degradation and the release of purified DNA. 

PCR amplification of HPV products used degenerate primers specific for the consensus regions of the L1 gene in the HPV genome. The assay was performed in two parts: HPV detection (presence/absence) and HPV identification (RFLP genotyping). The first reaction included two unique and specific primers, one for the HPV genome, and the other for the *β*-globin housekeeping gene. The latter was used as an internal control to document the presence of human nucleated cells in the sample analyzed. PCR products were then simultaneously separated and stained for visualization using 3% agarose gels prestained with ethidium bromide. Electrophoresis was performed in 1X TBE buffer at ~95 V for 90 minutes. Images of each gel were digitally captured. HPV type was determined by RFLP. A PCR product from each HPV-positive sample underwent endonuclease digestion with restriction enzymes *Pst I, Rsa I, *and* Hae III. *The digestion products were separated using 5% polyacrylamide gel electrophoresis (PAGE). The resultant fragment band patterns determined the specific HPV types present in each sample. 

For this analysis, oncogenic “high-risk” (HR) HPV types included 16, 18, 26, 31, 33, 35, 39, 45, 51, 52, 53, 56, 58, 59, 66, 68, 73, and 82 [16, 17]. All other HPV types, including unknown types, were considered low risk (LR). 

### 2.5. Statistical Analyses

Among the 290 women eligible for the study, 246 completed self-sampling and the subsequent questionnaire. Of these, we included 242 women in the present analysis; the four excluded women did not collect adequate specimens for HPV testing. All data were managed and analyzed in SAS version 9.2 for Windows (SAS Institute Inc., Cary, NC, USA). Frequency distributions of HPV infection and HPV types were calculated for the entire sample and then stratified according cytology result (normal versus abnormal [ASC-US+]). We also estimated HPV prevalence by age group (18–30; 31–40; 41–50; 51–60; >60 years) according to risk class (HPV 16/18, other HR, and LR types). To determine factors associated with prevalent HR HPV infection, we calculated odds ratios (ORs) and 95% confidence intervals with unconditional logistic regression models (univariate and adjusted for age). The reference group consisted of women with LR or no HPV. Women with multiple infections were included in the HR HPV group if at least one infection was with a HR HPV type.

## 3. Results

The vast majority of women (85.1%) were over 30 years of age (Table 1). Most participants (97.1%) were not born in the United States, but 74% had been in the USA for more than 5 years. While 49.6% reported a regular place for receiving healthcare, only 14.9% had health insurance. A significant proportion of women (79.3%) reported having had a Pap smear in their lifetime and 60.3% had been screened within the past 3 years. Approximately half (52.5%) of the sample were married or cohabitating, were employed part or full-time (55.8%), and reported more than 3 pregnancies in their lifetime (49.6%). Inflammation was present in cytological specimens for 45.9% of women, and the prevalence of vaginal infections with candida, gardnerella, or trichomoniasis was 33.1% (data not shown).

Table 2 reports the overall rates of HPV infection and prevalence by type among participants. Of the 50 women infected with HPV (20.7%), 8 were infected with multiple HPV types (3.3% of all women, 16% of HPV-positive women). HR HPV infections (13.2%) were considerably more common than LR infections (7.4%) among all women. The most prevalent HPV types, detected as either single or multiple infections, were HPV 53, 82, and 61 (each detected in 5 women). HPV 16 or 18 were detected in 5 specimens. Two samples were HPV positive but could not be genotyped and were subsequently classified as LR.

Overall, 21 women (8.7%) had abnormal cytology results, including 14 with atypical cells of undetermined significance (ASC-US), 1 with atypical glandular cells not otherwise specified (AGC-NOS), 4 with low-grade intraepithelial lesions (LSIL), and 2 with high-grade intraepithelial lesions (Table 3). Of women with abnormal cytology results, 16/21 (76.1%) had detectable HPV; of women with normal cytology results, 34/221 were HPV-positive (15.3%). The most common HPV types among women with abnormal cytology were HPV 82, 35, and 61 (each found in 2 women). The two women with HSIL were infected with HPV 35 and 82. There was no association between cytology result and risk class of HPV (*P* = 0.88).

**Figure 2:**
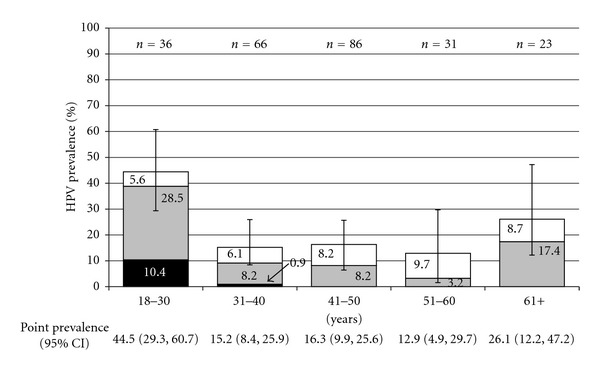
Age-specific prevalence of cervical human papillomavirus (HPV) DNA by LR, HR, and HR Types 16 and 18. *Vertical bars* indicated 95% confidence intervals of overall HPV prevalence. (Black) HPV 16 and/or 18 (including co-infection), (grey) all other HR-HPV, (white) LR-HPV only.

Figure 2 shows the prevalence of HPV (by HPV 16 or 18, other HR HPV, and LR types) across age strata. The age-specific prevalence of HPV decreased from 44.5% (95% CI: 29.360.7%) in those aged 18–30 to 15.2% (95% CI: 8.4–25.9%) among women 31–40, 16.4% (95% CI: 9.9–25.6%) in women 41–50, and 12.9% (95% CI: 4.9–29.7%) in 51–60 year olds. Prevalence increased to 26.1% (95% CI: 12.2–47.2%) among women aged 61 years and over. Similarly, the prevalence of HR HPV infections was highest in 18–30 year olds (38.9%), falling to 9.1%, 8.2%, and 3.2% in women aged 31–40 years, 41–50 years, and 51–60 years, respectively, and then increasing to 17.4% among women 61 years and over. By contrast, the prevalence of LR infections was lowest in 18–30 year olds (5.6%) and highest in older women (9.7% in 51–60 year olds). HPV 16 and 18 were found exclusively among women between 18 and 40 years, with 5 out of 6 infections occurring in women aged 30 and under. HR HPV infections outnumbered LR infections in the youngest and oldest age groups, while LR infections were at least as prevalent as HR infections in women between 41 and 60 years.

Table 4 shows the crude and age-adjusted ORs from logistic regression models for prevalent infection with HR HPV and the major characteristics of women in the study. In univariate analyses, younger age, never being married, being born outside the USA, never having been pregnant, and ever being exposed to tobacco were significantly associated with HR HPV infection. However, when we adjusted for age group, only ever exposure to tobacco smoke at home remained significantly associated with HR HPV infection (OR = 4.05, 95% CI: 1.2–12.8; *P* < .01). There was a low prevalence of concomitant sexually transmitted infections (STIs) in our sample including 13 cases of Chlamydia and 1 case of Gonorrhea. There was no significant association between STI infection and any HPV infection, HR HPV infection, or abnormal cytology. There were no significant associations between HR HPV positivity and vaginal infection.

## 4. Discussion

These data are the first to estimate the prevalence of HPV and distribution of infections by type and age in Haitian immigrants living in the USA. The crude prevalence of any HPV (20.7%) and HR HPV (13.2%) in this sample was slightly lower than estimates from a representative sample of USA women aged 15–49 years who provided self-collected HPV specimens (any HPV: 26.8%; HR HPV: 15.2%) [18]. Our estimates of any or HR HPV prevalence, both overall and for ages 60 and under, tended to be slightly lower than those reported by other USA-based studies [19–22]. Differences in study populations, statistical uncertainty, and HPV sampling and detection methods likely account for subtle differences.

We found that HR HPV types 53, 82, 52, and 68 and LR HPV types 61 and 62 were the most prevalent in the study population. This type distribution contrasts with findings from a meta-analysis of other USA-based studies, which found HPV 16, 52, 18, 51, and 58 to be the most prevalent types among women with normal cytology [23]. While the prevalence of HPV 16 tends to increase with the degree of cytologic abnormality (both in the USA and worldwide) [24], we found a relatively low prevalence of HPV 16 (1.2%), and none of these infections were associated with abnormal cytology. Cancer registry data aggregates individuals by broad race-based classifications and ethnicity data is often lacking and incomplete. Therefore, the HPV type distribution among cervical cancer cases in the Haitian immigrant population of Miami is unknown. The distribution of HPV types among women in Little Haiti bares similarity to study results from Dunne and colleagues [18], which also found HPV 53 to be the most prevalent HR HPV type in a representative USA sample, followed by HPV 52; HPV 62 was the most prevalent LR type. While we used a different self-sampling device than Dunne et al. and the Fournier device used in our study was designed to improve collection of cervical cells while minimizing sampling from the vagina, it is possible that the similar respective type distributions are an artifact of self-sampling. However, a study of paired vaginal and cervical specimens did not find significant differences in prevalence of HR HPV types (including the most prevalent ones in our study) based on sampling location; differences in HPV type distribution between cervical and vaginal specimens are more likely to arise among LR types in phylogenetic groups *α*3 (which includes HPV 61 and 62) and *α*15 [25]. Thus, the relatively high prevalence of HR HPV 53, 82, and 68 among our study population relative to meta-analysis of other USA studies would not appear to be a function of inadvertent sampling of vaginal cells. 

Data on the type distribution of HPV in Haiti is currently unavailable [26], although we are collecting in-country data that will allow for estimation of HPV burden and comparison between Haitian immigrants in the U.S. and women living in Haiti. Elsewhere in the Caribbean, overall HPV prevalence is higher than in our study. HPV 45 appears to be the most common HR type in Jamaica and Tobago [27, 28], while HPV 52 is the most prevalent HR type in Trinidad [29]. Geographical differences in the relative prevalence of HPV types—among Caribbean countries and the United States—may be influenced by an interaction between HPV types and host immunogenetic profile (for instance, human leukocyte antigen [HLA] polymorphisms) [24, 30]. HPV 16, the most oncogenic HPV type, appears to evade immune surveillance more effectively than other types [31]. Impairment of cellular immunity in a population (through immunocompromise or cervical inflammation, for instance) could thus lead to higher relative prevalence of HR types other than HPV 16 [24]. Haitian women routinely practice feminine hygiene with a wide-variety of solutions containing natural and commercial products. The very high frequency of cleansing, 2-3 times daily for most women, may be causing inflammation and disrupting the immune function of cervical cells. We note that the high level of cervical inflammation in our study (45.9%) may contribute to the high prevalence of non-16 HR types.

Age patterns associated with HPV prevalence vary by population. The prevalence of HPV among Haitian women living in Miami was highest in young women (44.5%), decreasing with age until rising to a second minor peak in women over 60 years (26.1%). A recent meta-analysis suggests that HPV prevalence in the USA declines steadily with age [23]. Data from Latin America and Central America, on the other hand, indicate a U-shaped curve, with prevalence declining through middle age but increasing again in older women [32–34]. The high prevalence of HR HPV among women over 60 years in our study population (17.4%) is cause for concern, particularly in light of low screening uptake. Prevalently detected infections in older women tend to represent persistent infections with an elevated risk of progression to cervical intraepithelial neoplasia grade 2 and higher (CIN2+) [35]. Our findings highlight the importance of improving screening coverage among older women in Little Haiti.

We found slightly elevated levels of cytological abnormalities (8.7%) compared to approximately 6% in the general USA population [36]. Still, few studies have examined the validity of cytological results obtained from samples self-collected with the Fournier device [13], so we interpret this finding with caution.

There are several limitations to this study. Our original study's planned sample size (300) was calculated based on the 95% exact confidence interval approach and good precision (i.e., less than 6%). In our study the observed prevalence of HPV for 242 evaluable women is 20.7% with 95% exact confidence interval of 15.7% and 26.3%. Therefore, our study established with high confidence (97.5%) that the true HPV prevalence in the target population is not less than 15.7%. Our study size is sufficiently large to ensure good precision, that is, a 5.3% semiwidth confidence interval, for estimating the HPV prevalence in this population. The sample size was reduced as a result of budgetary restraints and input from key members of the community advisory board. As a part of an ongoing CBPR initiative, it was necessary to balance the achievement of recruitment goals against the logistical constraints imposed by utilizing CHWs, who held roles in both this research study and as employees of various other community-based organizations.

Our sample size was small and when we stratified by age group we lost power to detect significant predictors of HR HPV infection. While age and exposure to tobacco smoke at home were significantly associated with HR HPV infection among our sample, the significance of other predictors was difficult to discern after adjusting for age. Furthermore, this was not a population-based sample, and results may not be fully generalizable to women living in Little Haiti. Nearly 80% of participants in this study reported receiving a Pap smear in the past three years, which is considerably higher than the 44% indicated by our previous work with women in Little Haiti [3]. We attribute the higher utilization of screening in the current study population to the community partners' unwillingness to limit inclusion to women who had never been screened in their lifetime. Participant selection may be a limitation of the study; CBPR relies upon community participation to define the focus of research and identify culturally appropriate recruitment strategies and methods of data collection. Our partnership with the community-based organization, and the affiliated CHWs, was critical to the high participation rate achieved. We encountered few barriers to study implementation and were able to build organizational capacity to support future research and intervention. Importantly, we were also able to ensure that data could be generated by the community and for the community's benefit.

Due to cultural considerations raised by our partner community-based organization, we could only collect data on broad age groups, and were limited in our ability to collect data on sexual behavior. Both age and sexual behavior are demonstrated predictors of HPV infection, and our inability to adjust for these in multivariate analysis is a limitation. We also rely on validity assessments of the self-sampling method from other studies, as we were not able to compare self-collected specimens to physician-collected specimens here. We cannot rule out the possibility that women may not have self-sampled correctly.

This cross-sectional study is a preliminary investigation into the prevalence and distribution of HPV types among women in Little Haiti, Miami, Florida. Among participants, the burden of vaccine types 6, 11, 16, and 18 was low, but further study will be needed to determine the prevalence of HPV 16 and 18 in Haitian immigrants with cervical cancer. Although the prevalence of HPV and HR HPV does not appear to be elevated among women in Little Haiti relative to the general USA population, we note the unusually high prevalence of HR HPV among women over 60 years, which may indicate a substantial number of persistent infections at high risk of progression to CIN2+. Underutilization of screening is undoubtedly a contributing factor to the high incidence of cervical cancer, but further study is needed to explain the excess cervical cancer burden in this population relative to others in South Florida. We are presently examining common feminine hygiene practices in this community of Haitian immigrants that may physiologically alter the cervix. Changes induced by exposure to particular compounds may reduce a woman's ability to clear infections, placing women at a greater risk of persistent HR HPV infection.

Self-collection of HPV specimens has been demonstrated to be acceptable and feasible among women in Little Haiti [2], and the present study points to the need to address screening disparities in this population, particularly among older women. We hope that the findings we present here catalyze further research and the movement of resources toward implementation and scale up of screening programs tailored to underserved populations in the United States.

## Figures and Tables

**Table 1:** Characteristics of 242 cervical self-sampling participants.

		*N*	%
Age	18–30	36	14.9
	31–40	66	27.3
	41–50	86	35.5
	51–60	31	12.8
	>60	22	9.1
Education	< High school	119	49.2
	High school	49	20.3
	> High school	74	30.6
Years in USA	<5	63	26.0
	6–10	82	33.9
	>10	97	40.1
Marital Status	Never been married	71	29.3
	Married/living with partner	127	52.5
	Divorced/widowed/separated	44	18.2
	Employed	134	55.8
	Unemployed	95	39.6
	Homemaker/Student/Other	11	4.6
Income <15 K		121	50.0
Have health insurance		36	14.9
Have a regular place for healthcare		120	49.6
Read/Speak Creole Only		98	40.7

		Mean	SD

Number of pregnancies		3.8	2.6
Age at first pregnancy		22.9	5.1

**Table 2:** Cytology results and type-specific HPV prevalence among 242 residents of Little Haiti.

Cytology results		Normal	Abnormal*
		*n* (% of row total)	
Total		221 (91.3%)	21 (8.7 %)
HPV −		187 (97.4%)	5 (2.6%)
HPV +		34 (68%)	16 (32%)
HR HPV +		22 (69%)	10 (31%)
LR HPV +/ Unknown		12 (67%)	6 (33%)

HR infections	Type	No. of infected women	
	53	4	0
	82	1	2
	52	3	0
	68	2	1
	16	2	0
	35	1	2
	18	1	1
	33	2	0
	45	2	0
	66	0	1
	31	0	1
LR infections			
	61	2	2
	62	0	0
	83	2	1
	CP108	1	0
	11	1	1
	72	1	0
	84	1	0
	Unknown	2	0
	42	0	1
	44	0	1

Multiple HR infections		4	2
	16 & 84	1	0
	52 & 53	0	1
	62 & 66	1	0
	62 & 68	1	0
	82 & CP108	1	1
Multiple LR infections		2	0
	62 & 61	1	0
	62 & 72	1	0

*Abnormal cytology includes the following diagnoses: ASC-US (*n* = 14), AGS-NOS (*n* = 1), LSIL (*n *= 4), and HSIL (CIS, CIN2, and moderate dysplasia, *n *= 2).

**Table 3:** Distribution of HR HPV^¥^ types and cytology results.

Total HR HPV positive women		32 (13.2%)	

Type	No. of infected women *n* (%)	Abnormal (*n* = 10)	Normal (*n* = 22)
16	2 (5.2)	0	2
18	2 (5.2)	1	1
31	1 (2.6)	1	0
33	2 (5.2)	0	2
35	3 (7.9)	2	1
45	2 (5.2)	0	2
52	3 (7.9)	0	3
53	4 (10.5)	0	4
66	1 (2.6)	1	0
68	3 (7.9)	1	2
82	3 (7.9)	2	1
HR coinfections*	6 (15.8)	2	4

			*n*/total (%)
Differential cytology results	ASC-US	6	6/14 (42.8)
among HR-HPV positive	LSIL	1	1/4 (25)
women	HSIL	1	1/2 (50)

^¥^HPV: human papillomavirus, HR: high risk (oncogenic HPV type).

*Coinfections by types 16 & 84, 52 & 53, 62 & 66, 62 & 68, (*n* = 1 for each listed) and CP6108 & 82 (*n *= 2).

**Table 4:** Crude and age-adjusted odds ratio estimates of HR HPV infection to negative and LR HPV infection (*n *= 242).

Age		%	Crude OR^¥^ (95% CI)	*P* value	Age-adjusted *OR (95% CI)	*P* value
	18–30	14.9	1			
	31–40	27.3	**0.16 (0.05–0.51)**		—	—
	41–50	35.5	**0.14 (0.04–0.43)**		—	—
	51–60	12.8	**0.05 (0.01–0.41)**		—	—
	>60	9.1	0.34 (0.07–1.32)	<.001	—	—
Educational attainment						
	< High school graduate	49.2	1		1	
	=> High school graduate	50.8	1.5 (0.70–3.17)	0.3	1.04 (0.38–2.88)	1
Marital status						
	Never married	29.3	1		1	
	Married/cohabitating	52.5	**0.39 (0.18–0.86)**		0.63 (0.24–1.65)	
	Divorced/Widowed/Separated	18.2	**0.07 (0.01–0.58)**	<.01	0.15 (0.01–1.15)	0.12
Place of birth						
	US born	2.9	1		1	
	Born abroad	97.1	**5.33 (1.14–25.0)**	<.001	1.22 (0.15–8.8)	1
Years in the United States						
	<5 years	26.0	1		1	
	5–10 yrs	33.9	0.73 (0.30–1.82)		1.02 (0.35–3.07)	
	>10 years	40.1	0.54 (0.22–1.37)	0.43	1.05 (0.33–3.36)	1
Employment status (*n *= 240)						
	Unemployed	39.6	1		1	
	Employed part or full time	55.8	1.19 (0.53–2.64)		1.27 (0.50–3.35)	
	Homemaker/Student/Other	4.6	2.86 (0.66–12.4)	0.37	1.19 (0.16–7.01)	0.90
Menopause status						
	Premenopausal	68.6	1		1	
	Postmenopausal	31.4	2.17 (0.85–5.51)	0.08	2.68 (0.47–29.8)	0.31
Number of pregnancies						
	None	9.5	1		1	
	1–3	40.9	**0.24 (0.09–0.65)**		0.34 (0.1–1.19)	
	>3	49.6	**0.14 (0.05–0.41)**	<.01	0.29 (0.07–1.26)	0.10
Age at first pregnancy (*n *= 215)						
	Under 18	12.1	1		1	
	18–25	51.6	1.88 (0.40–8.76)		2.67 (0.51–27.5)	
	26–41	36.3	1.0 (0.19–5.3)	0.38	1.61 (0.24–19)	0.33
Ever tobacco use						
	Nonsmoker	86.8	1		1	
	Former or current smoker	13.2	0.93 (0.30–2.85)	0.90	0.71 (0.16–2.5)	0.78
Ever exposed to tobacco smoke at home						
	Nonexposed		1		1	
	Exposed	10.3	**3.11 (1.18–8.21)**	0.03	**4.05 (1.2–12.8)**	<.01
Health insurance (*n *= 241)						
	None	85.1	1		1	
	Yes	14.9	1.07 (0.38–3.0)	0.90	0.76 (0.20–2.43)	0.79
Regular place for healthcare						
	None	50.4	1		1	
	Yes	49.6	1.02 (0.48–2.15)	0.96	1.06 (0.44–2.56)	1
Inflammation						
	Absent	54.1	1			
	Present	45.9	0.78 (0.37–1.67)	0.52	0.96 (0.38–2.41)	1
Any STI**						
	Absent	94.6	1		1	
	Present	5.4	1.21 (0.26–5.7)	0.82	0.68 (0.12–3.69)	0.72
Vaginal infection^‡^						
	T. Vaginalis	9.5	0.98 (0.28–3.52)	0.98	0.87 (0.14–3.74)	1
	Gardnerella	20.2	1.66 (0.72–3.87)	0.24	1.40 (0.50–3.65)	0.6
	Candida spp.	7.4	0.81 (0.18–3.69)	0.78	0.77 (0.08–4.1)	1
	Multiple Vag. Infection	4.1	0.72 (0.09–5.9)	0.72	0.89 (0.2–7.9)	1
	Any Vag. Infection	33.1	1.46 (0.68–3.13)	0.33	1.18 (0.47–2.85)	0.84

^¥^Likelihood ratio test *P*-value; bolded values indicate a significant difference comparing response to reference level at *P* < 0.05.

*OR (95% CI): Age-adjusted Odds ratio and 95% confidence interval; reported *P*-value from exact method and Score test.

**Includes HIV/AIDS (*n *= 0), Gonorrhea (*n *= 1), and Chlamydia (*n *= 13).

^‡^Same woman can be counted more than once due to multiple infections; OR is odds HR HPV infection for women with specific vaginal infection compared to those without.
